# Maternal voluntary wheel running modulates glucose homeostasis, the gut microbiota and its derived fecal metabolites in offspring

**DOI:** 10.1042/CS20230372

**Published:** 2023-08-09

**Authors:** Ling Zhang, Wenyu Zou, Yongyan Hu, Honghua Wu, Ying Gao, Junqing Zhang, Jia Zheng

**Affiliations:** 1 Department of Endocrinology, Peking University First Hospital, Beijing, China; 2 Laboratory Animal Facility, Peking University First Hospital, Beijing 100034, China

**Keywords:** glucose metabolism, gut microbiota, intestinal gluconeogenesis, maternal exercise, metabolomics

## Abstract

Maternal overnutrition can dramatically increase the susceptibility of offspring to metabolic diseases, whereas maternal exercise may improve glucose metabolism in offspring. However, the underlying mechanism programming the intergenerational effects of maternal exercise on the benefits of glucose metabolism has not been fully elaborated. C57BL/6 female mice were randomly assigned to four subgroups according to a diet and exercise paradigm before and during pregnancy as follows: NC (fed with normal chow diet and sedentary), NCEx (fed with normal chow diet and running), HF (fed with high-fat diet and sedentary), and HFEx (fed with high-fat diet and running). Integrative 16S rDNA sequencing and mass spectrometry-based metabolite profiling were synchronously performed to characterize the effects of maternal exercise on the gut microbiota composition and metabolite alterations in offspring. Maternal exercise, acting as a natural pharmaceutical intervention, prevented deleterious effects on glucose metabolism in offspring. 16S rDNA sequencing revealed remarkable changes in the gut microbiota composition in offspring. Metabolic profiling indicated multiple altered metabolites, which were enriched in butanoate metabolism signaling in offspring. We further found that maternal exercise could mediate gene expression related to intestinal gluconeogenesis in offspring. In conclusion, our study indicated that maternal running significantly improved glucose metabolism in offspring and counteracted the detrimental effects of maternal high-fat feeding before and during pregnancy. We further demonstrated that maternal voluntary wheel running could integratively program the gut microbiota composition and fecal metabolite changes and then regulate butanoate metabolism and mediate intestinal gluconeogenesis in offspring.

## Introduction

The prevalence of obesity and diabetes is growing rapidly worldwide [1], and substantial evidence supports that the intrauterine environment can significantly determine the susceptibility to developing obesity and diabetes in adult life [2]. This intergenerational phenomenon is known as the Developmental Origins of Health and Disease (DOHaD) hypothesis, which has been widely accepted and clearly established in recent years [3]. Our previous studies also suggested that maternal malnutrition, including overnutrition and undernutrition, could result in obesity, impaired glucose tolerance, and decreased insulin sensitivity in offspring [4–7]. Moreover, women of reproductive age with obesity and diabetes can launch a vicious cycle, which can propagate the risks of metabolic diseases to the next generation [8]. Thus, early life intervention during the critical window is warranted for preventing this deleterious transmission of obesity and diabetes.

Considering the particularity of pregnancy, a safe, feasible, and effective intervention measure should be proposed. Regular physical exercise, as a doctor’s natural, no-pill prescription for better health, has been well established to have whole-body benefits for people with obesity and diabetes [9]. Importantly, emerging human epidemiology studies suggest that physical exercise during pregnancy can improve adverse pregnancy outcomes in mothers and infants, including decreasing the incidence of gestational diabetes mellitus, macrosomia and stillbirth [10]. Several animal studies, including our previous findings, have demonstrated that exercise performed by dams before and during gestation could ameliorate glucose intolerance, insulin resistance and dyslipidemia in offspring [11–13]. However, the underlying mechanism programming the intergenerational effects of maternal exercise on the benefits of glucose metabolism has not been fully elaborated. It is an interesting question how physical exercise, as a transient stimulus during early development, can have long-term metabolic benefits in adult life.

Previous studies, including our findings, have shown that most tissues, including the liver [14], skeletal muscle [15], pancreas [11], and adipose tissue [16], in offspring could be imprinted by maternal exercise. However, little information is available concerning the gut microbiota in the beneficial effects of maternal exercise on glucose metabolism in offspring, given the critical role of the gut microbiota in obesity and diabetes [17,18]. The gut microbiota is proposed to serve a long-lasting memory function of the neonatal environment [19]. Our previous studies provide evidence supporting that maternal malnutrition can cause microbiota dysbiosis and metabolic disorders in offspring [7,20,21]. In addition, it showed that maternal obesity during pregnancy is associated with alterations in the diversity of the intestine microbial community, which can affect the gut microbiota composition in offspring [22,23]. It also demonstrated that the gut microbiota in infants of obese mothers increases inflammation and susceptibility to non-alcoholic fatty liver disease (NAFLD), supporting a causative role of maternal obesity-associated infant dysbiosis in childhood obesity and NAFLD [24]. This, it indicates the critical role of gut microbiota underlying maternal–offspring interaction.

Exercise, as a metabolic stimulus, can alter gut microbiota structures and composition that can benefit the glucose metabolism of the host [25]. Limited studies have examined the effects of maternal exercise on the gut microbiota in offspring [26–28]. However, the data are conflicting and are very descriptive, without further investigations about the exact function of the gut microbiota in offspring. The mechanisms underlying the programming effects of maternal exercise training on the gut microbiota in offspring are not yet well elaborated, and little is known about the cross-talk between the alterations in the gut microbiota and fecal metabolome profiling in offspring. In this study, we used a mouse model to determine whether maternal voluntary wheel running before and during pregnancy could attenuate the deleterious effects of maternal high-fat feeding on the long-term glucose metabolism of offspring. Moreover, integrative 16S rDNA-based microbial sequencing and mass spectrometry-based metabolic profiling were synchronously performed to characterize the programming effects of maternal exercise training on the gut microbiota composition and metabolite changes in offspring. Based on the comprehensive analysis of the microbial sequencing and metabolite profiling, we further deciphered the mechanisms by which the gut microbiota can mediate glucose homeostasis in offspring mice, including the gut microbiota-derived metabolites and intestinal gluconeogenesis.

## Materials and methods

### Mice and exercise paradigm

**Figure 1:**
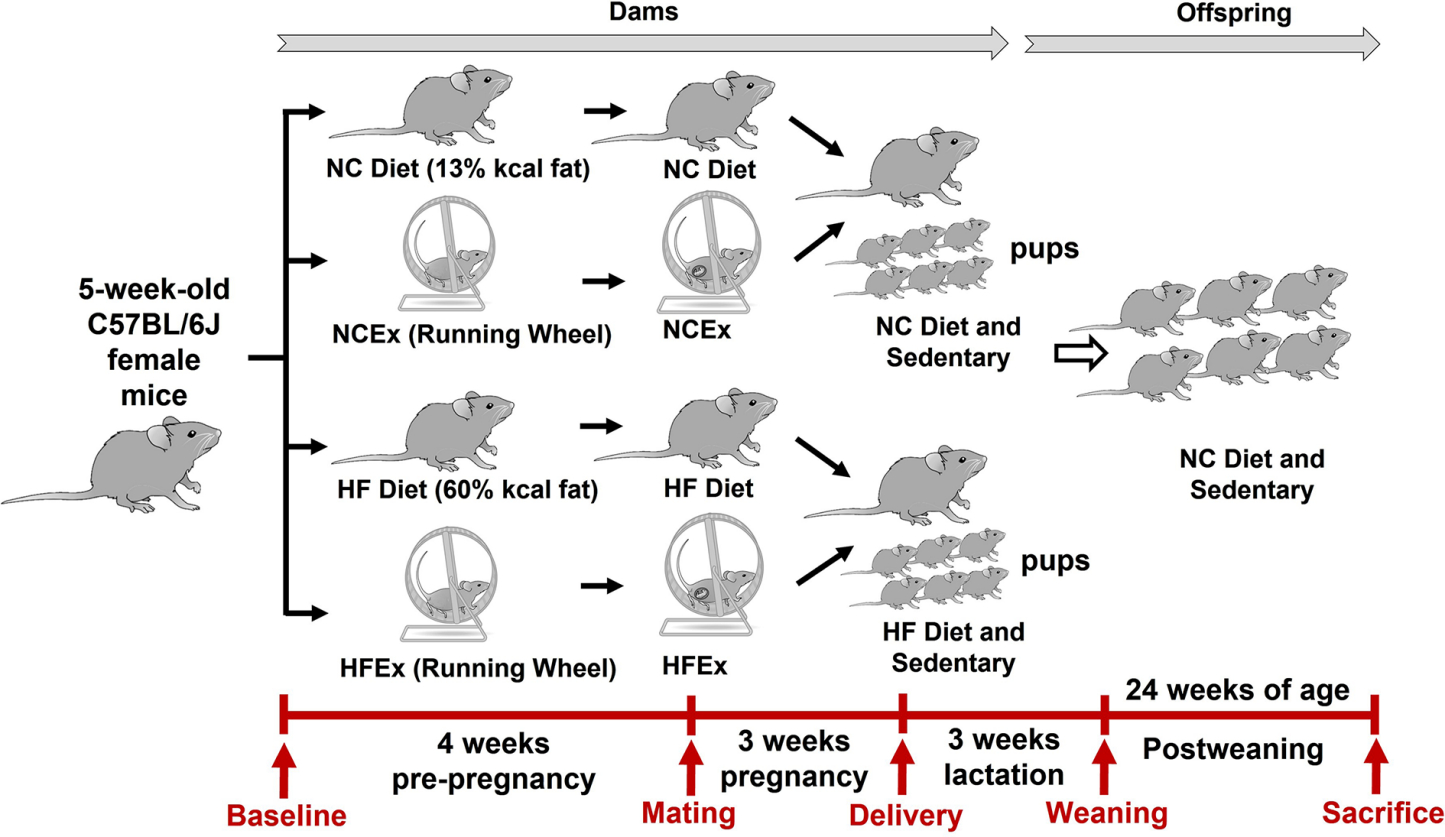
Experimental scheme of mice diet and exercise paradigm Ex, exercise; HF, high fat; HF group, dams fed with HF diet and housed in static cage; HFEx group, dams fed with HF diet and housed with voluntary running wheels; NC, normal chow; NC group, dams fed with NC diet and housed in static cage; NCEx group, dams fed with NC diet and housed with voluntary running wheels.

Five-week-old C57BL/6J virgin female mice were housed under standard conditions with ad libitum access to food and water (12-h light/dark cycle; 22 ± 2°C). After one week of adaptation, all female mice were randomly assigned to four subgroups according to diet and exercise paradigm before and during pregnancy as follows: (1) NC group (fed with normal chow (NC) diet and housed in static cages) (NC diet: 13% fat, Keao Xieli Feed Co., Ltd., Beijing, China); (2) NCEx group (fed with NC diet and housed with voluntary running wheels); (3) HF group (fed with high-fat (HF) diet and housed in static cages) (HF diet: 60% fat; Keao Xieli Feed Co., Ltd., Beijing, China); or (4) HFEx group (fed with HF diet and housed with voluntary running wheels). The running wheels were purchased from Yuyan Instruments Company and were 13 cm in diameter and 6 cm in width (Shanghai, China). All C57BL/6 male breeders were fed the NC diet and housed in static cages. Harem breeding was performed to minimize any potential differences in sires. Litters were standardized to six mice to avoid nutritional bias among litters, and all offspring mice were fed the NC diet and were sedentary from weaning to 24 weeks of age. A schematic representation of the diet and exercise paradigm is shown in Figure 1.

Body weight and food intake in offspring mice from weaning to 24 weeks of age were periodically recorded. At 24 weeks of age, one male offspring from each litter was fasted overnight. The mice were anesthetized and euthanized with sodium pentobarbital (50 mg/kg, intraperitoneally). Blood samples were then collected by cardiac puncture. The cecal contents and ileum were immediately collected, snap frozen, and stored at −80°C for further analysis. In our study, one male offspring from each litter (6–10 litters/group) was randomly selected for further studies to avoid confounding factors associated with the hormone profile and the estrous cycle in female offspring. In addition, sexual dimorphism has been reported in previous studies [15,29].

All mice were housed and used at the Experimental Animal Research Center of the Peking University First Hospital. The study was approved by the Ethics Committee for Animal Experimentation of the Faculty of Peking University First Hospital (Approval No. J201944), and all procedures were performed in accordance with the Guide for the Care and Use of Laboratory Animals (NIH).

### Glucose, insulin and pyruvate tolerance tests

For intraperitoneal glucose tolerance tests (ipGTTs), mice were fasted for 12 h and intraperitoneally injected with glucose (2.0 g/kg body weight). For intraperitoneal insulin tolerance tests (ipITTs), mice were fasted for 6 h and intraperitoneally injected with insulin (1 unit/kg body weight) (Humulin, Eli Lilly and Company, Indianapolis, IN). For intraperitoneal pyruvate tolerance tests (ipPTTs), mice were fasted for 12 h and intraperitoneally injected with pyruvate (1.5 g/kg body weight). The tests were performed as previously described, with an interval of three days between each test [7,30]. Blood glucose was determined using a portable blood glucose meter (Contour TS, Bayer, Beijing, China) and blood from the tail was tested at baseline and 15, 30, 60, and 120 min after injection.

The trapezoid method was utilized to calculate the area under the curve (AUC) to evaluate the blood glucose response to the tolerance tests.

### 16S rRNA gene sequencing analysis

To characterize the programming effects of maternal exercise on the gut microbiota composition, 16S rRNA gene sequencing was performed as described in our previous study [7]. Microbial genomic DNA was extracted from the fecal contents of male offspring using a QIAamp DNA Stool Mini Kit (Qiagen, Hilden, Germany) according to the manufacturer’s protocol. The V3-V4 region of the 16S rRNA genes was amplified using the primers 338F-806R (338F, 5′-ACTCCTACGGGAGGCAGCAG-3′; 806R, 5′-GGACTACHVGGGTWTCTAAT-3′). The PCR products were purified using the AxyPrep DNA Gel Extraction Kit (Axygen Biosciences, Union City, CA, U.S.A.), according to the manufacturer’s instructions and then quantified using a Quantus™ Fluorometer quantitative system (Promega, U.S.A.). After purification and quantification, high-throughput sequencing was performed on an Illumina MiSeq PE300 platform (Illumina, San Diego, U.S.A.). All procedures were performed according to the protocols of the Majorbio® Bio-Pharm Technology Company (Shanghai, China).

Bioinformatics analysis of the gut microbiota was carried out according to our recent study [7]. Raw data were optimized, and the chimeras were identified with the Uparse software platform (http://drive5.com/uparse/). The optimized sequences were clustered into operational taxonomic units (OTUs) using UPARSE (version 7.0) with 97% similarity [31]. The taxonomic information was annotated based on the Ribosomal Database Project (RDP) classifier (https://sourceforge.net/projects/rdp-classi er/) by the Silva Database (https://www.arb-silva.de/) [32]. Mothur (https://www.mothur.org/wiki/Download_mothur) was used to calculate the α diversity of the gut microbiota community, including the Shannon, Simpson, Ace, and Chao indices [33]. β-Diversity was calculated by the Quantitative Insights Into Microbial Ecology (QIIME) platform (http://qiime.org/install/index.html) based on unweighted and weighted UniFrac distance metrics [34]. Differential species at different classification levels in offspring were determined by using the Wilcoxon rank sum test. Principal coordinate analysis (PCoA) was performed by R (v.3.3.1). Linear discriminant analysis (LDA) effect size (LEfSe, http://huttenhower.sph.harvard.edu/galaxy/root?tool_id=lefse_upload) was performed to identify the species abundance differences in offspring using the nonparametric Kruskal‒Wallis rank sum test. The metagenome function of the microbiota in offspring based on the Kyoto Encyclopedia of Genes and Genomes (KEGG) database was predicted by Phylogenetic Investigation of Communities by Reconstruction of Unobserved States (PICRUSt) [35]. Covariation between microbial taxonomy and metabolic parameters was performed by Spearman correlation analysis.

### Fecal metabolite profiling

Fecal metabolites in offspring mice were detected by liquid chromatography‒mass spectrometry (LC-MS) as previously described [36]. Briefly, 50 mg of cecal contents of each offspring mouse was prepared and processed for further chromatography and mass spectrometry. Chromatographic separation was performed on a 1.8 µm, 100 mm × 2.1 mm BEH C18 column (Waters Acquity™, U.S.A.). The mobile phases were 0.1% formic acid in water and acetonitrile/isopropanol (v/v, 1:1) with 0.1% formic acid. The flow rate was 0.4 ml/min, and mass spectrometry detection was performed by a triple TOF 5600+ MS/MS system (AB Sciex, Concord, Ontario, Canada). Positive- and negative-ion electrospray ionization modes were detected. The capillary and cone voltages were optimized at 2.5 kV and 40 V, respectively. Mass data were collected from the range of *m*/*z* 50–1500 in mass spectrometry elevated energy (MSE) continuum mode. Quality control (QC) samples were prepared to validate data quality.

For metabolomic analysis, the data were analyzed by the Majorbio Cloud platform (https://cloud.majorbio.com). The raw LC‒MS data were preprocessed using Waters Progenesis QI 2.0 software (Nonlinear Dynamics, Newcastle, U.K.). All the metabolites identified by LC‒MS were matched, and the annotation of metabolites was determined by the KEGG (http://www.genome.jp/kegg/) database and Human Metabolome Database (HMDB, www.hmdb.ca). The orthogonal partial least squares discriminant analysis (OPLS-DA) algorithm was utilized to visually compare the different metabolite profiles in offspring. The importance of each metabolite was ranked according to the variable importance in projection (VIP) scores, and KEGG pathway enrichment analysis was determined by R packages and Python software (with VIP score ≥1 and *P-*value <0.05). Covariation between differential fecal metabolites and metabolic parameters was performed by Spearman correlation analysis.

### Correlation analysis between microbial taxonomy and fecal metabolites in offspring

Procrustes analysis [37] was used to investigate the correlations between the 16S rRNA sequencing data and LC-MS-based metabolites in offspring mice. The correlation between the top 20 gut species based on richness and the top 20 differential metabolites according to VIP scores was analyzed by Spearman correlation analysis. The correlations between the gut microbiota and metabolites were analyzed by the hierarchical clustering algorithm and average hierarchical clustering method. The analysis was processed by the Majorbio Cloud platform (https://cloud.majorbio.com/).

### RNA extraction and quantitative RT-PCR analyses

Total RNA was extracted from ileum tissues using TRIzol reagent (Invitrogen, Waltham, MA, U.S.A.) and reverse-transcribed into cDNA using Reverse Transcription Kits (Thermo Fisher Scientific, NH, U.S.A.), and cDNA was amplified by the appropriate primers. The primer sequences are listed in Supplementary Table S1. Quantitative real-time PCR was performed on an ABI PRISM 7500 Detection System (Applied Biosystems, Life Technologies) with a SYBR Select Master Mix kit (Thermo Fisher Scientific, NH, U.S.A.). The cycle threshold (Ct) value was normalized to the housekeeping gene β-actin. All reactions were performed in replicates, and the 2^–∆∆Ct^ method was utilized to calculate the relative expression levels of genes.

### Statistical analysis

The data were analyzed using GraphPad Prism 9.0 software. The data are presented as the mean ± standard error (SEM). ANOVAs followed by Tukey’s post-hoc test were used for multiple comparison analysis. Data on the gut microbiota and metabolomic sequencing were analyzed according to the corresponding methods as previously mentioned. *P*<0.05 was considered statistically significant.

## Results

### Maternal voluntary wheel running before and during pregnancy prevented the detrimental effects of maternal HF feeding on glucose metabolism in offspring

**Figure 2:**
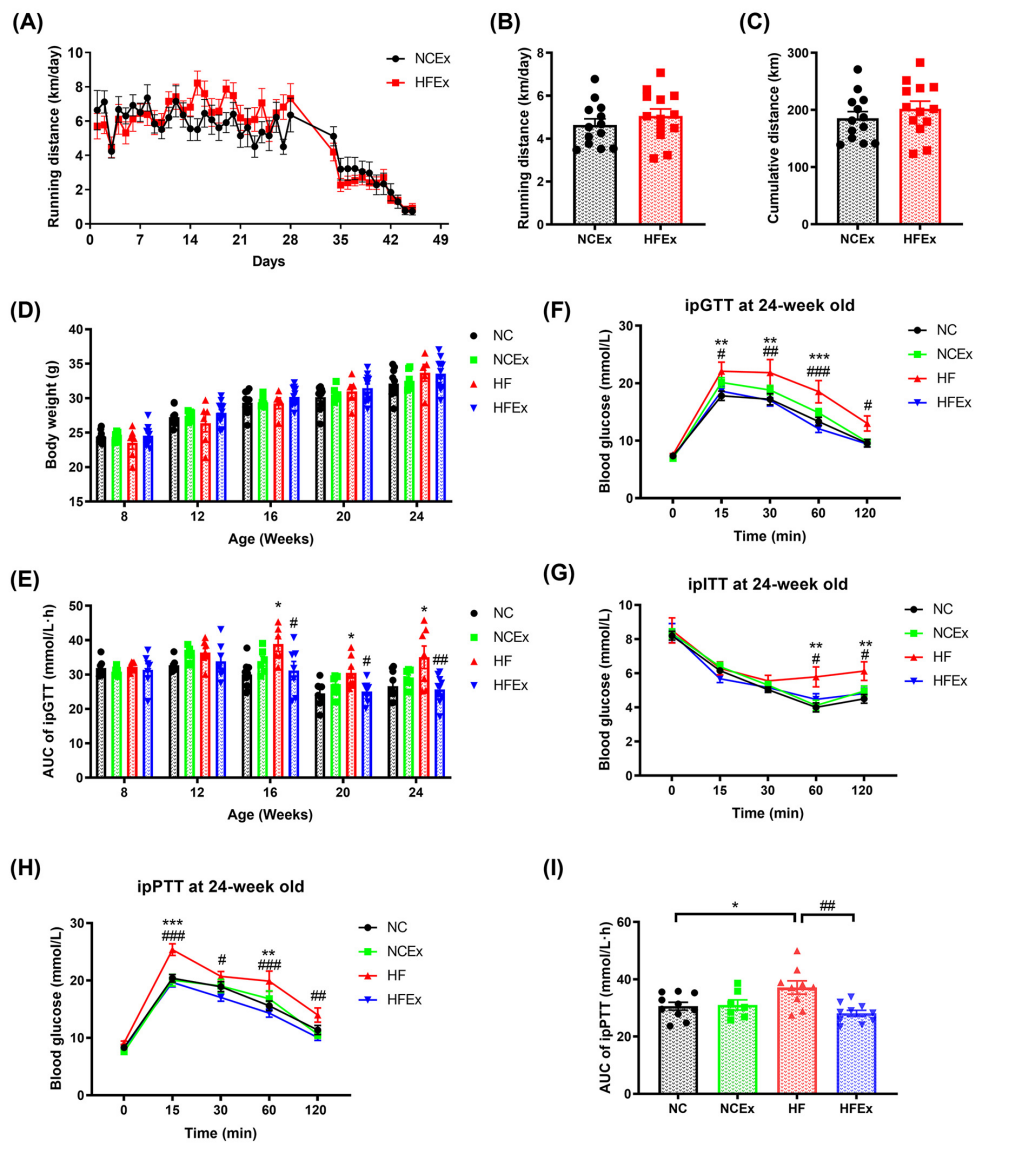
Maternal exercise ameliorated the detrimental effects of a maternal high-fat diet on offspring glucose metabolism (**A**) Running distance monitoring of dams during 4 weeks before mating and throughout pregnancy. (**B**) Average running distance per day of dams. (**C**) Cumulative running distance of dams during pre-pregnancy and pregnancy. (**D**) Body weight monitoring of offspring mice. (**E**) AUC of blood glucose values of ipGTTs of offspring from 8 to 24 weeks of age. (**F**) ipGTT of offspring at 24 weeks of age. (**G**) ipITT of offspring at 24 weeks of age. (**H**) ipPTT of offspring at 24 weeks of age. (**I**) AUC of blood glucose values of ipPTT. AUC, area under the curve; Ex, exercise; HF, high fat; HF group, offspring of dams fed with HF diet and housed in static cage; HFEx group, offspring of dams fed with HF diet and housed with voluntary running wheels; ipGTT, intraperitoneal glucose tolerance test; ipITT, intraperitoneal insulin tolerance test; ipPTT, intraperitoneal pyruvate tolerance test; NC, normal chow; NC group, offspring of dams fed with NC diet and housed in static cage; NCEx group, offspring of dams fed with NC diet and housed with voluntary running wheels. *n* = 6–10 litters/group, one male offspring per litter. *P<0.05, **P<0.01, ***P<0.001, HF vs. NC group; ^#^*P*<0.05, ^##^*P*<0.01, ^###^*P*<0.001 HFEx vs. HF group.

To determine whether maternal exercise can ameliorate the detrimental effects of maternal HF feeding, female mice were fed an NC or HF diet and housed in regular cages or cages with voluntary running wheels for 4 weeks before breeding and throughout pregnancy. There was no difference in body weight at baseline and before breeding of dams. Food intake of dams was measured weekly and corrected for spillage. It showed that no difference in average energy intake of dams was observed among the four groups (Supplementary Figure S1). The running distance of dams before and during pregnancy is shown in Figure 2A. The dams ran approximately 6 km per day before mating. As pregnancy progressed, the running distance of dams gradually decreased. As shown in Figure 2B, the average running distances of the dams fed the NC diet and HF diet were approximately 4.6 ± 0.3 km and 5.1 ± 0.3 km, respectively (*P*>0.05). There was no difference in the cumulative running distance between dams fed the NC and HF diets (*P*>0.05, Figure 2C).

Body weight was measured per week from weaning to 24 weeks of age. There was no significant difference in body weight among the four groups from 8 weeks of age to 24 weeks of age (*P*>0.05, Figure 2D). ipGTTs were performed dynamically at an interval of 4 weeks in male offspring mice. As shown in Figure 2E, sedentary dams fed a HF diet resulted in marked glucose intolerance in male offspring as they aged (*P*<0.05 at 16 weeks of age, *P*<0.01 at 20 weeks of age, and *P*<0.01 at 24 weeks of age). Offspring from dams fed a HF diet and housed with voluntary running wheels were protected from these deleterious effects of a maternal HF diet from 16 weeks of age, which persisted to 24 weeks of age (*P*<0.05 at 16 weeks of age, *P*<0.05 at 20 weeks of age, and *P*<0.01 at 24 weeks of age). Thus, the improvements in glucose tolerance in offspring from dams that voluntarily exercised before and during gestation were not correlated with the changes in body weight, demonstrating that improvements in glucose metabolism were not due to changes in body weight.

For ipGTTs in offspring at 24 weeks of age, the blood glucose levels of the male offspring of dams fed a HF diet and housed in static cages were significantly higher at 15 min (*P*<0.01), 30 min (*P*<0.01), and 60 min (*P*<0.001) compared with offspring mice of the NC group. Remarkably, the blood glucose levels of the male offspring from HF-fed and exercised dams were significantly decreased at 15 min (*P*<0.05), 30 min (*P*<0.01), 60 min (*P*<0.001), and 120 min (*P*<0.05) compared with those of the HF group (Figure 2F). An insulin tolerance test was further performed to detect insulin sensitivity in offspring mice. Insulin tolerance was impaired in offspring from HF-fed dams, with higher blood glucose levels at 60 min (*P*<0.01) and 120 min (*P*<0.01), and this effect was significantly attenuated in offspring from HF-fed and exercised dams, with lower blood glucose levels at 60 min (*P*<0.05) and 120 min (*P*<0.05) (Figure 2G). We further determined the capacity of liver gluconeogenesis by the pyruvate glucose test in offspring mice. Strikingly, the blood glucose levels of the male offspring of dams fed a HF diet and housed in static cages were significantly higher at 15 min (*P*<0.01) and 60 min (*P*<0.001) after intraperitoneal pyruvate administration than those of the NC group, and this effect was significantly attenuated in offspring of HF-fed and exercised dams, with lower blood glucose levels at 15 min (*P*<0.001), 30 min (*P*<0.01), 60 min (*P*<0.001), and 120 min (*P*<0.01) (Figure 2H). Consistently, the AUC of the pyruvate glucose test was also significantly decreased in the offspring of exercised dams (Figure 2I). These findings demonstrated that maternal HF feeding resulted in prominent glucose intolerance and insulin resistance in male offspring, whereas maternal voluntary wheel running before and during the pregnancy period can prevent these deleterious effects of maternal HF feeding.

### Maternal exercise altered the gut microbiota structure and composition in offspring

**Figure 3:**
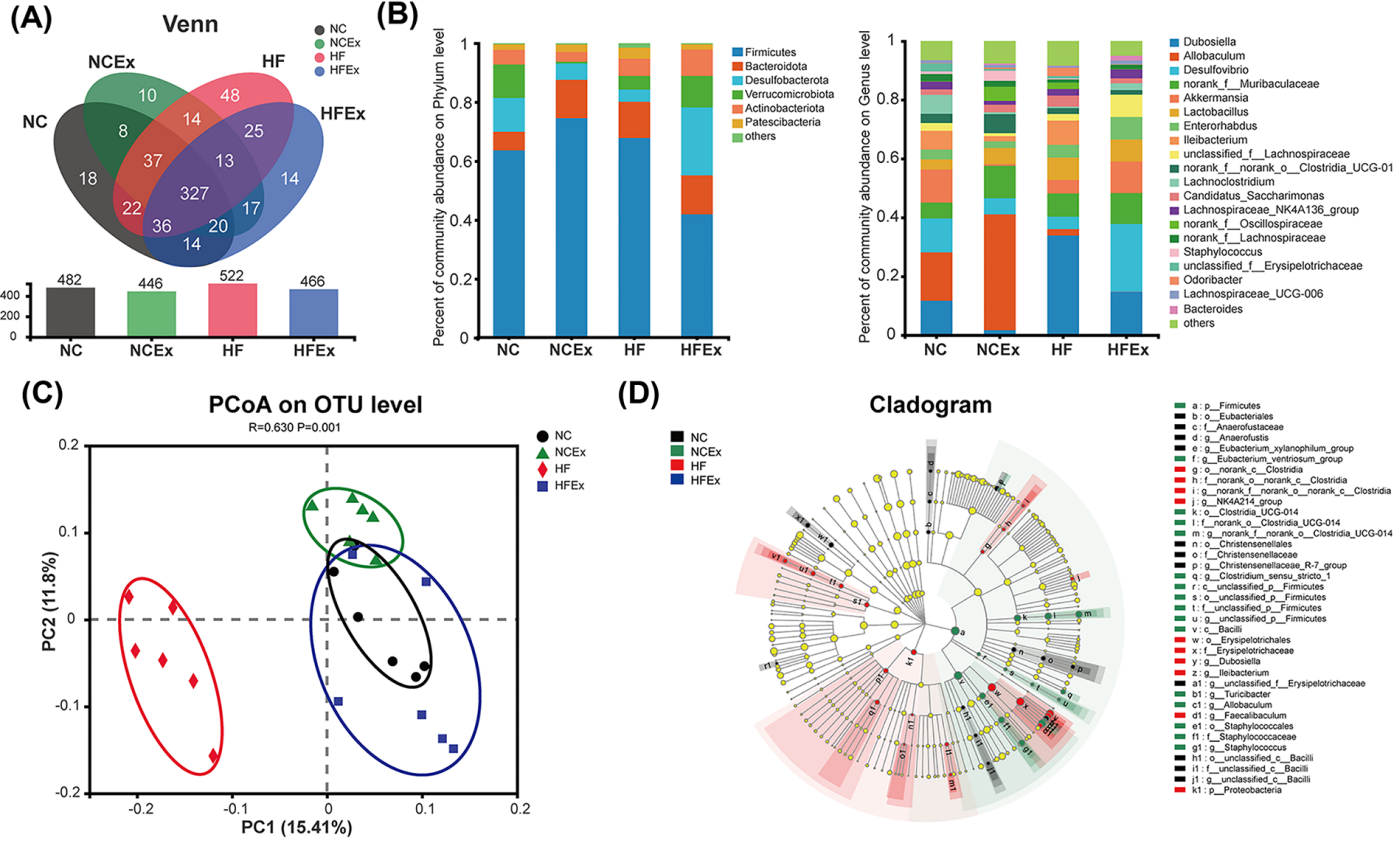
Maternal exercise altered structures and composition of gut microbiota in offspring (**A**) Venn diagram of the OUTs. (**B**) Relative abundance of the bacterial population at the phylum (B1) and genus (B2) levels. (**C**) PCoA plot of gut communities in four groups. (**D**) Taxonomic representation of statistically and biologically consistent differences among four groups. Differences are represented by the color of the most abundant class (black: NC group; green: NCEx group; red: HF group, blue: HFEx group). The diameter of each circle is proportional to the taxon’s abundance. Ex, exercise; HF, high fat; NC, normal chow; OTU, operational taxonomic unit; PCoA, principal coordinate analysis. *n* = 6 litters/group, one male offspring per litter.

To determine the effects of maternal exercise on the gut microbiota structure and composition in offspring, the 16S rRNA gene amplicon was sequenced in offspring. As shown in Figure 3A, shared and unique OTUs were analyzed in offspring mice. The results indicated 327 shared OTUs among the four groups, 100 unique OTUs in the offspring from HF-fed dams compared with offspring from NC-fed dams, and 65 unique OTUs in the offspring from dams fed a HF diet and housed with voluntary running wheels compared with offspring from HF-fed and sedentary dams. There were no significant differences in the α diversity of the gut microbiota community among the four groups, including the Shannon, Simpson, Ace, and Chao indices (Supplementary Table S2). Then, the relative abundances of the gut microbial composition at the phylum and genus levels were evaluated in offspring. At the phylum level, *Firmicutes, Bacteroidetes, Desulfobacterota, Verrucomicrobiota*, and *Actinobacteria* were the most abundant in offspring mice (Figure 3B1 and Supplementary Figure S2A). Compared with the NC group, the HF group had a higher abundance of *Bacteroidetes* and a lower abundance of *Desulfobacterota*. The abundance of *Firmicutes* was deceased, and the abundances of *Desulfobacterota* and *Verrucomicrobiota* were increased in offspring of HF-fed and exercised dams compared with offspring from HF-fed and sedentary dams. It shows the top 20 species at the genus level in offspring mice (Figure 3B2 and Supplementary Figure S2B). The PCoA plot revealed significantly different community distributions among the four groups (*R* = 0.63, *P*=0.001) (Figure 3C). A cladogram indicated significant differences in the phylogenetic distributions among the four groups, which represented the taxonomic hierarchy from phylum to species (Figure 3D). These results indicated that maternal exercise can result in remarkable changes in the gut microbiota composition in offspring.

**Figure 4:**
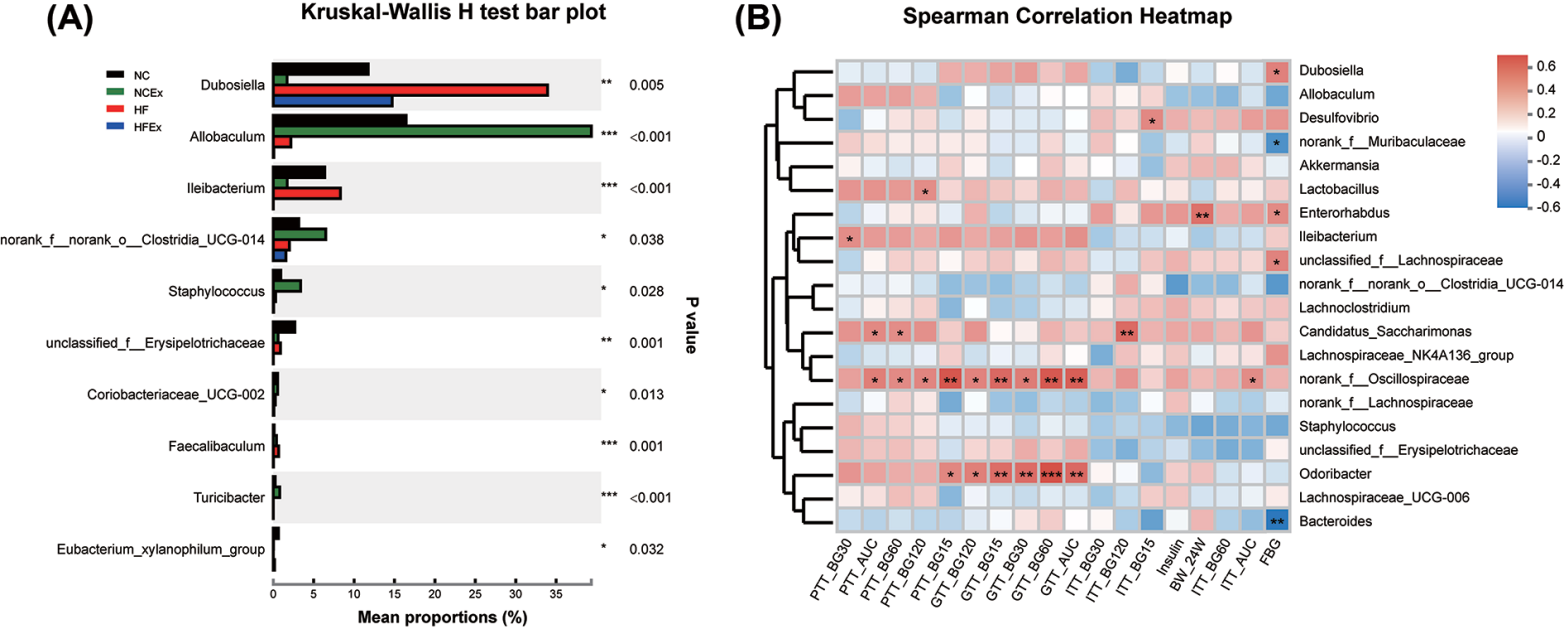
Differential species at the genus level and the correlation between the gut microflora and metabolic parameters in offspring (**A**) Column chart of top 10 species with significant differences at the genus level in offspring. (**B**) Heatmap of correlation analysis between the differential genera and metabolic parameters in offspring. The bacteria were on the right, and the metabolic parameters were at the bottom. Each grid represented the correlation between the two attributes, with the red-positive correlation, and the blue-negative correlation. AUC, area under the curve; BW_24W, body weight of 24 weeks of age; Ex, exercise; FBG, fasting blood glucose; GTT_AUC, AUC of ipGTT; GTT_BG15, 30, 60, and 120: blood glucose levels at 15, 30, 60, 120 min of ipGTTs; HF, high fat; ipGTT, intraperitoneal glucose tolerance test; ipITT, intraperitoneal insulin tolerance test; ipPTT, intraperitoneal pyruvate tolerance test; ITT_AUC, AUC of ipITT; ITT_BG15, 30, 60, and 120: blood glucose levels at 15, 30, 60, 120 min of ipITTs; NC, normal chow; PTT_AUC, AUC of ipPTT; PTT_BG15, 30, 60, and 120: blood glucose levels at 15, 30, 60, 120 of ipPTTs. *n* = 6 litters/group, one male offspring per litter; *P<0.05, **P<0.01, ***P<0.001.

To further examine the alterations in microbiota composition, we analyzed the differential species in offspring using the Wilcoxon rank sum test. The top 10 genera whose abundances were significantly different among the offspring were *Dubosiella, Allobaculum, Ileibacterium, norank_f_norank_o_Clostridia_UCG-014, Staphylococcus, unclassified_f__Erysipelotrichaceae, Coriobacteriaceae_UCG-002, Faecalibaculum, Turicibacter*, and *Eubacterium_xylanophilum_group*. Most of the differential gut microbiota in offspring was reported to produce short-chain fatty acids (SCFAs) (Figure 4A and Supplementary Table S3).

### Functional predictions of the bacterial communities by the KEGG pathway database in offspring

To predict the functional metagenomic profiles of bacteria, KEGG pathway analysis was performed as level 1—six category pathways, level 2—subcategory pathways, and level 3—secondary pathways. The KEGG pathway level 1 analysis showed pathways related to metabolism, genetic information processing, environmental information processing, cellular processes, human diseases, and organismal systems (Supplementary Table S4). KEGG pathway level 2 indicated that six pathways were significantly enriched, namely, carbohydrate metabolism, metabolism of cofactors and vitamins, metabolism of terpenoids and polyketides, cancer: specific types, infectious disease: viral, and circulatory system (Supplementary Table S4). KEGG pathway level 3 exhibited significant differences in 72 metabolic functions. Of note, endocrine and metabolic disease and lipid metabolism pathways, such as Type 2 diabetes mellitus and fatty acid degradation, were significantly altered by maternal exercise (Supplementary Table S4).

### Correlation analyses between the gut microbiota and metabolic parameters in offspring

We further examined the correlation between the top 20 genera and metabolic parameters in offspring mice by Spearman correlation analysis. The metabolic parameters included body weight, serum insulin, blood glucose and the AUCs of the ipGTTs, ipITTs, and ipPTTs. Interestingly, we found that most of the bacteria were positively correlated with the blood glucose levels in the ipGTTs, ipITTs, and ipPTTs, including *norank_f_Oscillospiraceae, Odoribacter, Candidatus_Saccharimonas, Enterorhabdus*, and *Ileibacterium*. The abundance of *Enterorhabdus* was positively correlated with body weight. The abundances of *Bacteroides* and *norank_f_Muribaculaceae* were negatively associated with blood glucose in the pyruvate tolerance test (Figure 4B).

### Maternal exercise programed fecal metabolite changes in offspring

**Figure 5:**
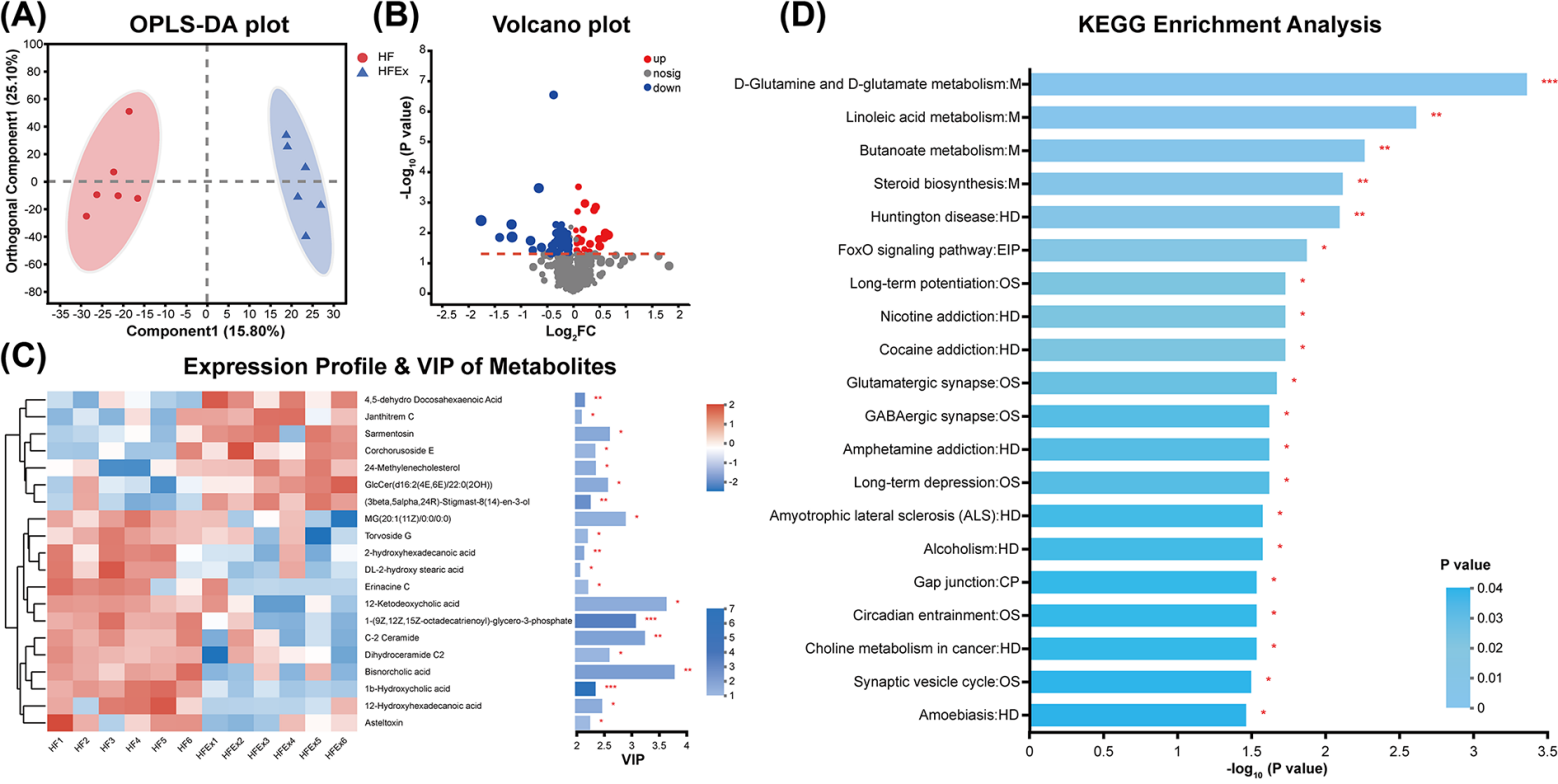
Alterations in the fecal metabolic profile in offspring (**A**) OPLS-DA plots showing spatial division between offspring mice. Red dot denotes each mouse of HF group and blue dot denotes each mouse of HFEx group. (**B**) The volcano plot graph of altered metabolites in offspring. (**C**) Hierarchical clustering and heatmap in the left panel showing the top 20 metabolites that were significantly differentially abundant in offspring. Each row represents data for a specific metabolite, and each column represents one mouse. Different colors correspond to different metabolite abundance levels. Red and blue colors represent increased and decreased levels of metabolites, respectively. The histogram in the right panel represents variable VIP scores derived from the OPLS-DA model for each metabolite. (**D**) Top 20 KEGG pathway enrichment analysis. The *X*-axis represents the metabolites significance of enrichment (−log_10_(*P-*value)) in pathway, the *Y-*axis represented the impact value of metabolites in the pathway. *n* = 6 litters/group, one male offspring per litter. *P<0.05, **P<0.01, and ***P<0.001. CP, cellular processes; EIP, Environmental Information Processing; Ex, exercise; HD, human diseases; HF, high fat; M, metabolism; OS, organismal systems; VIP, importance in projection.

To further understand the microbial responses to bacterial perturbations in offspring mice, fecal metabolome characterization was performed to identify metabolites that discriminate between offspring of HF-fed and exercised dams compared with offspring from HF-fed and sedentary dams. First, an OPLS-DA model was constructed, and the OPLS-DA score plot exhibited distinct discrimination between the two groups (R^2^X (cum) = 0.409, R^2^Y (cum) = 0.975, Q^2^ (cum) = 0.738), indicating that the OPLS-DA model was reliable and predictive. This result suggested that the differences in the abundances of metabolites were highly significant between offspring mice of the HFEx and HF groups (Figure 5A). Hierarchical clustering was utilized to identify the differential metabolites with VIP scores>1 and *P*<0.05. Finally, 76 differentially abundant metabolites were identified between the HFEx and HF groups, with 28 metabolites that were increased and 48 metabolites that were decreased (Figure 5B). A heatmap was constructed to visualize the top 20 differentially abundant metabolites according to VIP score (Figure 5C). Seven metabolites were significantly higher in offspring mice of the HFEx group: 4,5-dehydro docosahexaenoic acid, janthitrem C, sarmentosin, corchorusoside E, 24-methylenecholesterol, GlcCer(d16:2(4E,6E)/22:0(2OH)), and (3beta,5alpha,24R)-stigmast-8(14)-en-3-ol. Thirteen metabolites were significantly lower in offspring mice of the HFEx group: MG(20:1(11Z)/0:0/0:0), torvoside G, 2-hydroxyhexadecanoic acid, DL-2-hydroxy stearic acid, erinacine C, 12-ketodeoxycholic acid, 1-(9Z,12Z,15Z-octadecatrienoyl)-glycero-3-phosphate, C-2 ceramide, dihydroceramide C2, bisnorcholic acid, 1b-hydroxycholic acid, 12-hydroxyhexadecanoic acid, and asteltoxin.

### Functional predictions of fecal metabolites by the KEGG pathway database in offspring

To predict the metabolic function of fecal metabolites, metabolic pathway enrichment was performed according to the KEGG database (Figure 5D). Compared with the HF group, 20 pathways were enriched in the offspring of HF-fed and exercised dams, with four pathways related to metabolism (M), eight pathways related to human diseases (HD), one pathway related to environmental information processing (EIP), six pathways related to organismal systems (OS), and one pathway related to cellular processes (CP). Notably, the top three enriched pathways included D-glutamine and D-glutamate metabolism, linoleic acid metabolism, and butanoate metabolism.

### Correlation analyses between the differential metabolites and metabolic parameters in offspring

**Figure 6:**
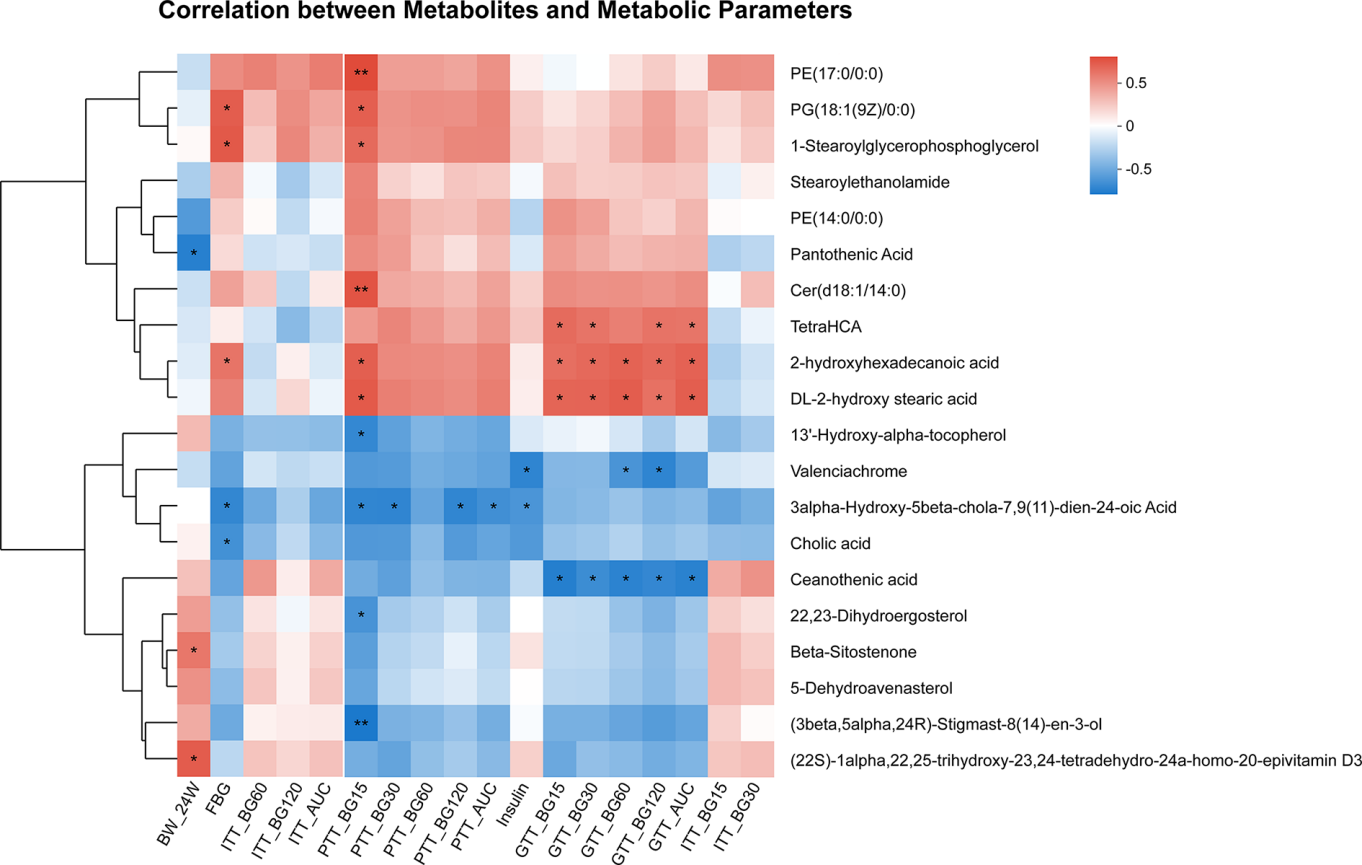
Heatmap of correlation analysis between the differential metabolites and metabolic parameters in offspring The metabolites were on the right, and the metabolic parameters were at the bottom. Each grid represented the correlation between the two attributes, with the red-positive correlation, and the blue-negative correlation. AUC, area under the curve; BW_24W, body weight of 24 weeks of age; Ex, exercise; FBG, fasting blood glucose; GTT_AUC, AUC of ipGTT; GTT_BG15, 30, 60, and 120: blood glucose levels at 15, 30, 60, 120 min of ipGTTs; HF, high fat; ipGTT, intraperitoneal glucose tolerance test; ipITT, intraperitoneal insulin tolerance test; ipPTT, intraperitoneal pyruvate tolerance test; ITT_AUC, AUC of ipITT; ITT_BG15, 30, 60, and 120: blood glucose levels at 15, 30, 60, and 120 min of ipITTs; NC, normal chow; PTT_AUC, AUC of ipPTT; PTT_BG15, 30, 60, and 120: blood glucose levels at 15, 30, 60, and 120 of ipPTTs. *n* = 6 litters/group, one male offspring per litter; *P<0.05, **P<0.01, ***P<0.001.

We further examined the correlations between the top 20 metabolites and metabolic parameters in offspring by Spearman correlation analysis. The included metabolic parameters were as mentioned above. Most of the metabolites were correlated with blood glucose and the AUCs of the ipGTTs, ipITTs, and ipPTTs. Notably, Cer(d18:1/14:0), tetraHCA, 2-hydroxyhexadecanoic acid, and DL-2-hydroxy stearic acid were positively related to blood glucose. Valenciachrome, 3α-hydroxy-5β-chola-7,9(11)-dien-24-oic acid, and ceanothenic acid were negatively related to blood glucose (Figure 6).

### Correlation between the intestinal microflora and metabolites

**Figure 7:**
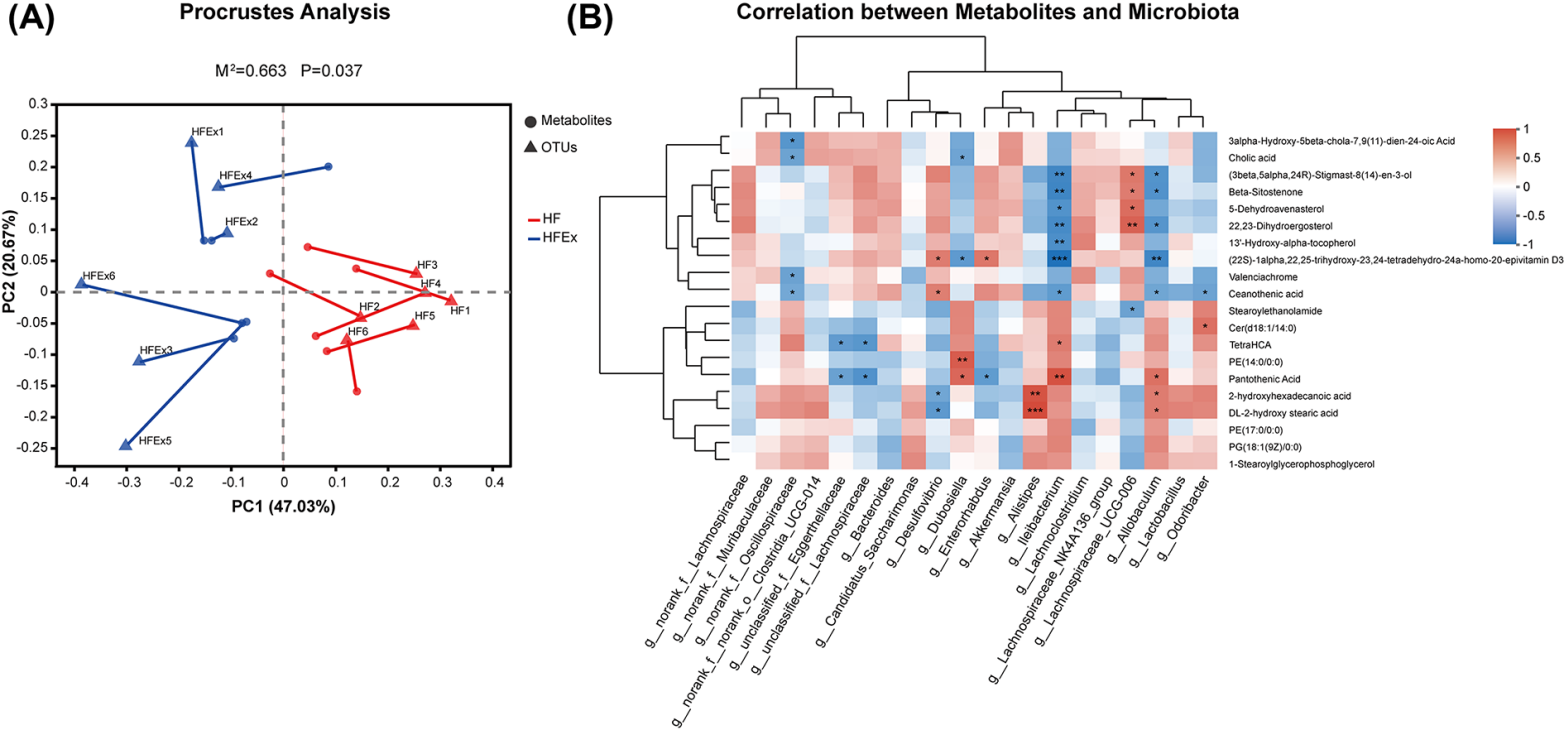
Correlation between intestinal microflora and metabolites in offspring mice (**A**) Procrustes analysis of intestinal microflora and metabolites. Red and blue colors indicate HF and HFEX groups, respectively. *M*^2^ is the goodness-of-t statistic of the two ordering results in Procrustes analysis, which is used to evaluate the correlation between the two sorting results. The *P-*value is the result of Monte Carlo permutation test, which is used to test the significance of *M*^2^. (**B**) Heatmap of correlation analysis between the differential metabolites and intestinal microflora. Correlation matrix between top 20 VIP differential metabolites and top 20 enriched bacteria. The metabolites were on the right, and the bacteria were at the bottom. Each grid represented the correlation between the two attributes, with the red-positive correlation, and the blue-negative correlation. n = 6 litters/group, one male offspring per litter. *P<0.05, **P<0.01, ***P<0.001. Ex, exercise; HF, high fat; VIP, importance in projection.

Procrustes analysis was performed to investigate the correlations between the intestinal microflora and metabolites, indicating significant consistency between the intestinal microflora and fecal metabolites (*M*^2^ = 0.663, *P*=0.037). This result suggested that the changes in the intestinal microflora caused by maternal exercise were significantly correlated with the gut metabolites (Figure 7A). The correlation between the top 20 intestinal microflora and top 20 metabolites in offspring was analyzed by the Spearman correlation coefficient. A total of 17 metabolites were associated with 11 related genera, of which 6 belonged to the phylum Firmicutes, 2 belonged to the phylum Bacteroidetes, 2 belonged to the phylum Actinobacteriota, and 1 belonged to the phylum Desulfobacterota. Remarkably, the abundances of two bacteria that can produce SCFAs, namely, Ileibacterium and Allobaculum, were significantly correlated with most fecal metabolites (Figure 7B).

### Maternal exercise mediated intestinal gluconeogenesis in offspring

**Figure 8:**
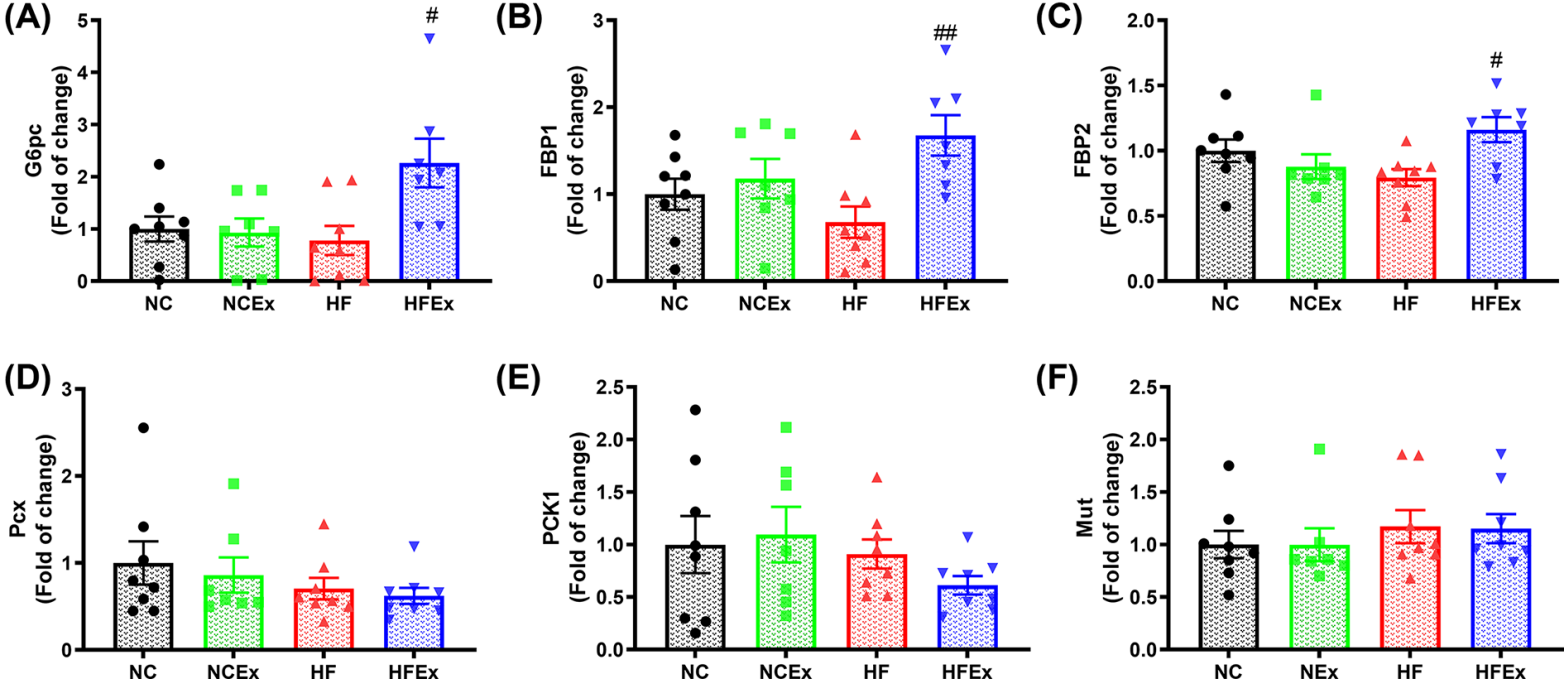
Maternal exercise mediated intestinal gluconeogenesis in offspring (**A–F**) mRNA expressions of core genes involved in the gluconeogenesis in the intestine, including G6pc, FBP1, FBP2, Pcx, PCK1, and Mut. FBP1, fructose-1,6-bisphosphatase 1; FBP2, fructose-1,6-bisphosphatase 2; G6pc, glucose 6-phosphate, catalytic; Mut, methylmalonyl-coA mutase; PCK1, phosphoenolpyruvate carboxykinase 1; Pcx, pyruvate carboxylase. *n* = 7–8 litters/group, one male offspring per litter. *P<0.05, **P<0.01, ***P<0.001, HF vs. NC group; ^#^*P*<0.05, ^##^*P*<0.01, ^###^*P*<0.001 HFEx vs. HF group. Ex, exercise; HF, high fat; NC, normal chow.

**Figure 9:**
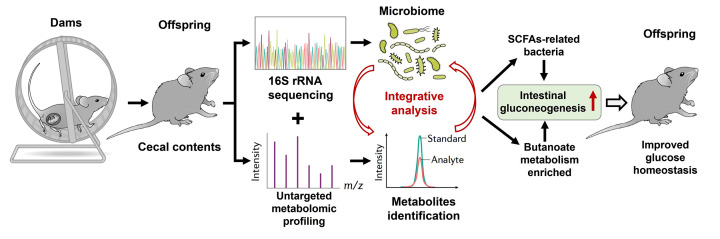
Working model A proposed view that maternal voluntary wheel running before and during pregnancy modulated gut microbiota composition and the metabolic changes, which enhanced the potency of intestinal gluconeogenesis and then improved glucose homeostasis in offspring. SCFAs, short-chain fatty acids.

Considering that most of the differential gut microbiota in offspring can produce SCFAs and that the top three enriched pathways of gut metabolites include butanoate metabolism, we further focused on the butanoate metabolism pathway. Butyrate, as an important SCFA, can exert critical physiological and pharmacological effects on glucose metabolism [38]. It has been demonstrated that butyrate can mediate intestinal gluconeogenesis, exhibiting protective effects against obesity and diabetes by decreasing hepatic glucose production and regulating glucose homeostasis [38,39]. To evaluate the effects of altered gut microbiota and metabolites on intestinal gluconeogenesis, we measured the mRNA expression of core genes involved in gluconeogenesis in the ileum, including glucose 6-phosphate, catalytic subunit (G6pc), fructose-1,6-bisphosphatase 1 (FBP1), fructose-1,6-bisphosphatase 2 (FBP2), pyruvate carboxylase (Pcx), phosphoenolpyruvate carboxykinase 1 (PCK1), and methylmalonyl-CoA mutase (Mut). The mRNA expression levels of FBP1 (*P*<0.01), FBP2 (*P*<0.05), and G6pc (*P*<0.05) in the intestine were significantly increased in the offspring of HF-fed and exercised dams compared with those of sedentary dams (Figure 8A–F). Taken together, these data show that maternal voluntary wheel running before and during pregnancy can modulate the gut microbiota composition and fecal metabolite changes in offspring, which enhances the potency of intestinal gluconeogenesis and improves glucose homeostasis in offspring (Figure 9).

## Discussion

Diabetes is a complex disease arising from both environmental and genetic factors and is increasing dramatically worldwide. It has become well established that risk patterns for diabetes can originate from intrauterine malnutrition in early life development [2]. It has long been recognized that exercise has important health benefits for obese and diabetic patients, and regular physical exercise can distinctly postpone or prevent the incidence of obesity and diabetes [40]. Clinical studies have indicated that maternal physical exercise before and during pregnancy can significantly decrease adverse pregnancy outcomes [41, 42]. Our study found that maternal voluntary wheel running before and during gestation enhanced glucose tolerance and decreased insulin resistance, suggesting an improvement in whole-body glucose homeostasis, which is consistent with previous studies [14–16]. These data demonstrate that maternal exercise is critical to improve the glucose metabolism of adult offspring.

There may be adaptations to numerous changes in the offspring that mediate the detrimental effects of maternal poor nutrition on glucose metabolism and the beneficial effects of maternal exercise in offspring. Previous studies have demonstrated that maternal exercise can program glucose metabolism in several tissues in offspring, including the liver [14], skeletal muscle [15], adipose tissue [16], and pancreas [11]. Considering the distinct effects of maternal exercise on glucose tolerance in the offspring and the increasingly pivotal role of the gut microbiota in glucose homeostasis, we hypothesized that the gut microbiota and its derived fecal metabolites could also be adapted in the offspring. Thus, integrative 16S rDNA sequencing and gut metabolite profiling were originally performed to explore the mechanisms by which maternal voluntary wheel running exerts beneficial effects on glucose homeostasis in offspring. Indeed, we found that maternal voluntary wheel running before and during pregnancy altered the gut microbiota composition and programmed fecal metabolite changes in offspring.

Maternal HF feeding resulted in a lower relative abundance of *Eubacterium_xylanophilum_group*, which was partially recovered in offspring of HF-fed dams that were exercised. Several studies have demonstrated that the *Eubacterium_xylanophilum_group* genus is associated with the production of SCFAs. This result indicated that *Eubacterium xylanophilum* could ferment complex phytochemicals to produce SCFAs, including butyrate [43]. Zhuge et al. recently demonstrated that probiotic-treated rats exhibited an enrichment in the *Eubacterium_xylanophilum_group* taxon that produces SCFAs, which played a role in mucoprotection and glucose metabolism [44]. In addition, the relative abundances of *Odoribacter* and *norank_f_Oscillospiraceae* were positively associated with blood glucose and PTTs in offspring. Consistently, *Odoribacter*, a genus belonging to the *Bacteroides* phylum, was reported to be highly abundant in db/db mice [45] and enriched in hypercholesterolemic subjects [46]. Huang et al. showed that *Odoribacter* was enriched in diabetic ICR mice and was positively related to the levels of body weight, insulin resistance, and glucagon-like peptide-1. Furthermore, the levels of *Odoribacter* were significantly decreased by treatment with one polysaccharide, which had hypoglycemic and hypolipidemic effects in T2DM mice [47]. In addition, *Oscillospiraceae*, a genus belonging to the *Firmicutes* phylum, was recently found to be significantly increased in HF-fed mice, and the relative abundance of *Oscillospiraceae* was significantly reduced in response to herbal medicine supplementation [48]. Collectively, these results supported that the *Odoribacter* and *norank_f_Oscillospiraceae* genera might result in impaired glucose metabolism in offspring mice.

Characterization of fecal metabolome changes can contribute to understanding the metabolic regulation of the gut microbiota perturbations. The fecal metabolite profiles were significantly different between offspring of HF-fed dams that were exercised and those that were sedentary. Remarkably, ceramide was significantly decreased in offspring from HF-fed and exercised dams. Ceramide, as the precursor of most sphingolipids, has been widely discovered to accumulate in individuals with obesity, insulin resistance, and dyslipidemia [49]. Similarly, recent data reported that ceramide could be modulated by diet and aerobic exercise [50], indicating that fecal ceramide changes in offspring may be regulated by maternal exercise. Notably, the *Ileibacterium* and *Allobaculum* genera, both belonging to the phylum *Firmicutes*, have been reported to produce SCFAs [51], and they were significantly correlated with most fecal metabolites. Thus, gut microbiota-derived fecal metabolites in offspring could be modulated by maternal exercise.

A striking finding was that most of the differential gut microbiota in offspring can produce SCFAs, and functional predictions of fecal metabolites by the KEGG pathway database were enriched in butanoate metabolism signaling. Butyrate is an important SCFA that is fermented by intestinal bacteria and plays a critical role in energy metabolism [52]. This finding demonstrates that butyrate can induce beneficial metabolic effects through activation of intestinal gluconeogenesis [38,39]. Intestinal gluconeogenesis is a recently described physiological phenomenon that positively regulates glucose and energy homoeostasis to exert antidiabetes and antiobesity effects. The induction of intestinal gluconeogenesis reduces hepatic glucose production and improves whole-body glucose homeostasis [38]. In our study, it demonstrated that maternal exercise could increase glucose tolerance and improve pyruvate tolerance in offspring. This improvement in glucose homeostasis is likely because of the higher abundance of SCFA-related bacteria and increased intestinal gluconeogenesis potency of adult offspring.

In conclusion, our study indicated that maternal voluntary wheel running before and during pregnancy significantly improved the glucose metabolic health of offspring and counteracted the detrimental effects of a maternal high-fat diet, including impaired glucose and puruvate tolerance, and decreased insulin sensitivity in offspring. To the best of our knowledge, our study is the first to demonstrate that maternal voluntary wheel running could integratively program the gut microbiota composition and fecal metabolite changes and then regulate butanoate metabolism and mediate intestinal gluconeogenesis in offspring. These findings can advance our thinking regarding the critical role of an altered gut microbiota and metabolites underlying the intergenerational effects of maternal exercise on glucose homeostasis in offspring [53].

## Clinical perspectives

- Maternal over-nutrition can dramatically increase the susceptibility of metabolic diseases in offspring, whereas maternal exercise may improve glucose metabolism in offspring. However, the underlying mechanism programming the intergenerational effects of maternal exercise on the benefits of glucose metabolism has not been fully elaborated.
- In the present study, we demonstrated that maternal voluntary wheel running could integratively programed gut microbiota composition and fecal metabolites changes, and then regulated butanoate metabolism and mediated intestinal gluconeogenesis in offspring.
- These findings can advance our thinking about the critical role of altered gut microbiota and metabolites underlying the intergenerational effects of maternal exercise on glucose homeostasis in offspring.

## Supplementary Material

Supplementary Figures S1-S2 and Tables S1-S4

## Data Availability

All supporting data are included within the main article and supplementary files. The raw data have been submitted to Figshare (DOI: 10.6084/m9.figshare.22699402, https://figshare.com/account/articles/22699402).

## Abbreviations

AUC: area under the curve
DOHaD: developmental origins of health and disease
FBG: fasting blood glucose
LDA: linear discriminant analysis
PCK1: phosphoenolpyruvate carboxykinase 1
SCFA: short-chain fatty acid
NAFLD: non-alcoholic fatty liver disease
OTUs: operational taxonomic units
FBP1: fructose-1,6-bisphosphatase 1
FBP2: fructose-1,6-bisphosphatase 2
PCx: pyruvate carboxylase
G6pc: glucose-6-phosphatase catalytic subunit
Mut: methylmalonyl-CoA mutase
ipGTT: intraperitoneal glucose tolerance test
ipPTT: intraperitoneal pyruvate tolerance test
ipITT: intraperitoneal insulin tolerance test
LC-MS: liquid chromatography‒mass spectrometry
RDP: Ribosomal Database Project
QIIME: Quantitative Insights Into Microbial Ecology
PCoA: Principal coordinate analysis
KEGG: Kyoto Encyclopedia of Genes and Genomes
LDA: Linear discriminant analysis
QC: Quality control
PICRUSt: Phylogenetic Investigation of Communities by Reconstruction of Unobserved States
MSE: mass spectrometry elevated energy
VIP: variable importance in projection
HMDB: Human Metabolome Database
OPLS-DA: orthogonal partial least squares discriminant analysis
