# Supplementary material for: Maternal voluntary wheel running modulates glucose homeostasis, the gut microbiota and its derived fecal metabolites in offspring
Source: Clin Sci (Lond). 2023 Aug 9;137(15):1151–66. doi: 10.1042/CS20230372 (PMC10412464; doi:10.1042/CS20230372)
Supplement: Supplementary Figures S1-S2 and Tables S1-S4 [file CS-2023-0372_supp.pdf]

Supplementary Figure

Fig. S1. Body weight, and food intake of dams, and body weight of offspring. (A) Maternal body weight at baseline and prepregnancy. **(B)** Maternal energy intake. **(C)** Body weight of offspring from 1 week of age to 3 weeks of age. n=11 litters/group, one male offspring per litter. *P < 0.05 , HF vs. NC group.

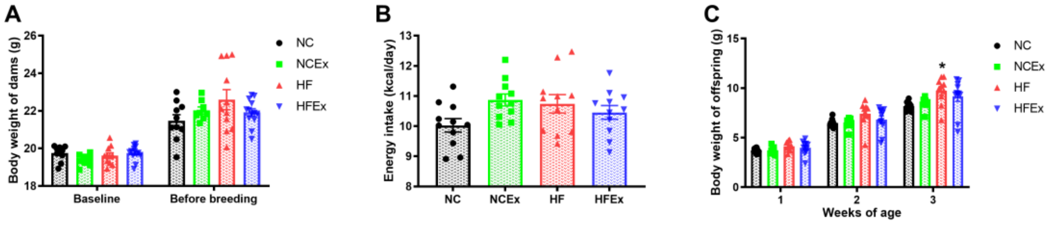

Fig. S2. Maternal exercise altered structures and composition of gut microbiota in offspring. (A) Relative abundance of the bacterial population at the phylum level of each mouse. **(B)** Relative abundance of the bacterial population at the genus level of each mouse.

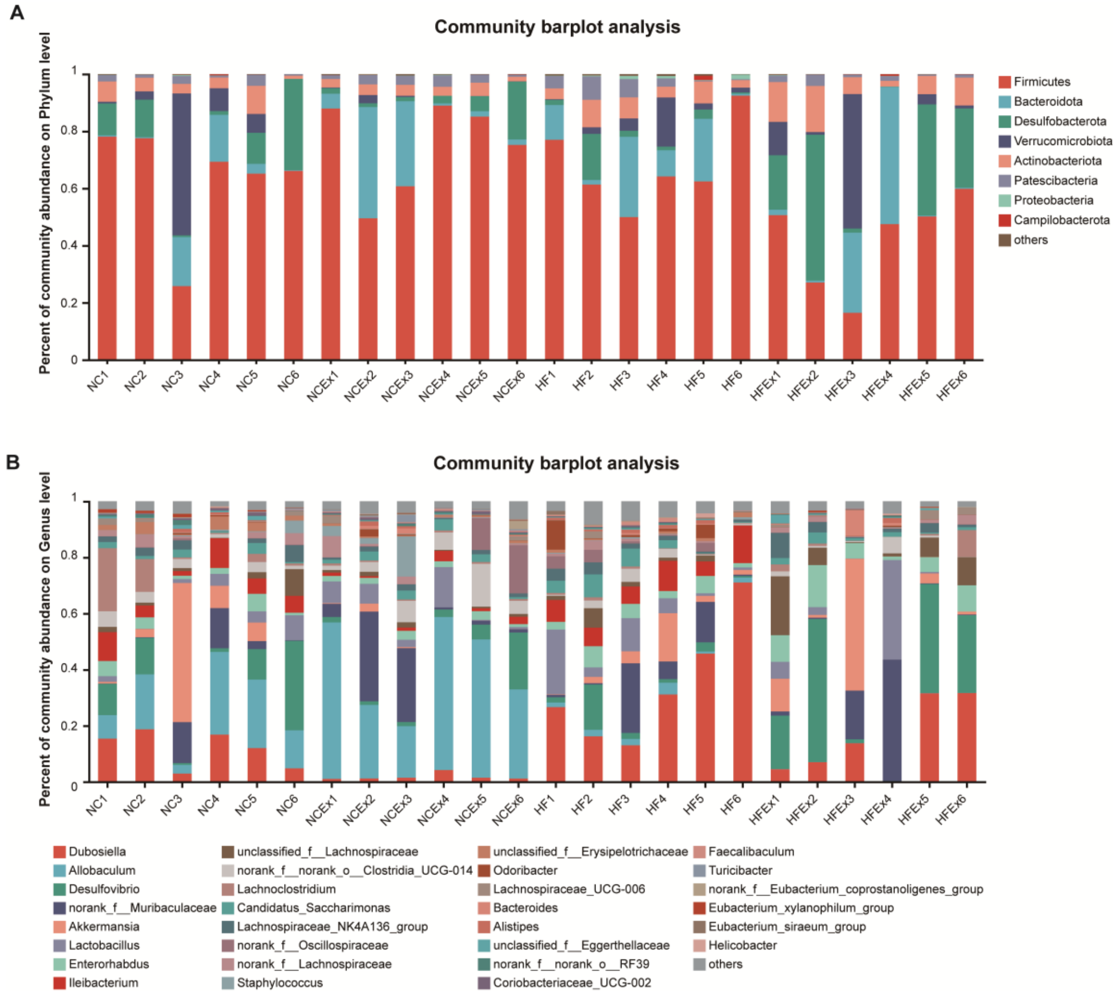

Supplementary Tables

Table S1. Primer sequences of genes.

Genes	Forward	Reverse
**G6Pc**	CGACTCGCTATCTCCAAGTGA	GTTGAACCAGTCTCCGACCA
**FBP1**	CACCGCGATCAAAGCCATCT	AGGTAGCGTAGGACGACTTCA
**FBP2**	ACCCTGACCCGTTACGTTATG	ACATTCACGCTCCCCGAAATC
**PCx**	CTGAAGTTCCAAACAGTTCGAGG	CGCACGAAACACTCGGATG
**PCK1**	CTGCATAACGGTCTGGACTTC	CAGCAACTGCCCGTACTCC
**Mut**	TTTTTGCTATCGCCCCATTACC	CCTCTGGGTTTTTGCCTTTCAG
**β-actin**	TATTGGCAACGAGCGGTTCC	GGCATAGAGGTCTTTACGGATGTC

G6P, glucose 6-phosphate; FBP1, fructose-1,6-bisphosphatase 1; FBP2, fructose-1,6-bisphosphatase 2; Pcx, pyruvate carboxylase; PCK1, phosphoenolpyruvate carboxykinase 1; Mut, methylmalonyl-coA mutase.

Table S2. Alpha diversity of gut microbiota community in offspring.

Index	NC	NCEx	HF	HFEx	P value (HF vs. NC)	P value (HFEx vs. HF)
**Shannon**	3.04±0.14	2.88±0.78	3.00±0.87	2.70±0.60	0.99	0.86
**Simpson**	0.13±0.06	0.20±0.12	0.18±0.18	0.19±0.09	0.89	0.99
**Ace**	348.60±31.27	344.40±28.60	365.90±49.89	320.80±33.69	0.85	0.18
**Chao**	340.60±33.22	341.60±28.03	362.40±51.92	327.10±36.70	0.76	0.41

Table S3. Differential species at the genus level among the four groups.

Species name	NC-Mean (%)	NCEx-Mean (%)	HF-Mean (%)	HFEx-Mean (%)	P value
g__Flavobacterium	0.000	0.000	0.014	0.000	0.000
g__Allorhizobium-Neorhizobium-Pararhizobium-Rhizobium	0.000	0.000	0.197	0.000	0.000
g__Roseomonas	0.000	0.000	0.015	0.000	0.000
g__Allobaculum	16.430	39.360	2.172	0.061	0.000
g__Turicibacter	0.230	0.807	0.000	0.001	0.000
g__Ileibacterium	6.374	1.733	8.314	0.012	0.000
g__Cyanobium_PCC-6307	0.000	0.000	0.007	0.000	0.000
g__Bifidobacterium	0.001	0.012	0.245	0.031	0.001
g__Faecalibaculum	0.113	0.391	0.667	0.031	0.001
g__unclassified_f__Erysipelotrichaceae	2.665	0.598	0.887	0.010	0.001
g__norank_f__T34	0.000	0.000	0.012	0.001	0.001
g__Limnohabitans	0.000	0.000	0.005	0.001	0.002
g__norank_f__Candidatus_Hepatincola	0.000	0.000	0.018	0.000	0.003
g__Dubosiella	11.750	1.716	33.940	14.720	0.005
g__Prevotellaceae_NK3B31_group	0.018	0.001	0.009	0.001	0.010

g__Eubacterium_ventriosum_group	0.017	0.101	0.000	0.002	0.012
g__Coriobacteriaceae_UCG-002	0.541	0.539	0.247	0.084	0.013
g__unclassified_p__Firmicutes	0.007	0.039	0.001	0.000	0.013
g__Pluralibacter	0.000	0.001	0.007	0.000	0.013
g__Herbaspirillum	0.000	0.001	0.004	0.000	0.013
g__Anaerofustis	0.107	0.017	0.009	0.020	0.018
g__NK4A214_group	0.023	0.016	0.087	0.003	0.019
g__Anaerovorax	0.003	0.000	0.020	0.000	0.019
g__Aeromonas	0.000	0.000	0.207	0.000	0.020
g__Fournierella	0.000	0.000	0.012	0.000	0.020
g__Staphylococcus	0.925	3.385	0.305	0.047	0.028
g__Burkholderia-Caballeronia-Paraburkholderia	0.002	0.007	0.033	0.008	0.028
g__Clostridium_sensu_stricto_1	0.117	0.372	0.006	0.001	0.032
g__Eubacterium_xylanophilum_group	0.636	0.033	0.009	0.156	0.032
g__unclassified_c__Bacilli	0.102	0.087	0.076	0.003	0.035
g__norank_f__norank_o__Clostridia_UCG-014	3.150	6.486	1.992	1.572	0.038
g__Christensenellaceae_R-7_group	0.320	0.031	0.168	0.060	0.038
g__norank_f__norank_o__norank_c__Clostridia	0.002	0.003	0.045	0.003	0.049

Table S4. Functional predictions of bacterial communities by KEGG pathway database in offspring.

	Name	P value	NC-Mean (%)	NCEx-Mean (%)	HF-Mean (%)	HFEx-Mean (%)
**KEGG-level 1**	Metabolism	0.964	76.590	76.710	76.520	76.850
	Genetic Information Processing	0.132	8.502	8.509	8.774	8.224
	Environmental Information Processing	0.867	6.267	6.261	6.202	6.293
	Cellular Processes	0.774	3.870	3.810	3.648	3.942
	Human Diseases	0.337	3.125	3.089	3.204	3.067
	Organismal Systems	0.704	1.643	1.621	1.649	1.625
**KEGG-level 2**	Carbohydrate metabolism	0.044	10.620	11.080	10.780	10.170
	Amino acid metabolism	0.963	6.997	7.009	7.017	6.937
	Energy metabolism	0.138	4.205	4.147	4.185	4.423
	Metabolism of cofactors and vitamins	0.012	3.726	3.557	3.475	4.003
	Lipid metabolism	0.650	1.731	1.810	1.728	1.775
	Biosynthesis of other secondary metabolites	0.082	1.551	1.480	1.557	1.568
	Glycan biosynthesis and metabolism	0.609	1.557	1.362	1.533	1.641

	Metabolism of other amino acids	0.311	1.182	1.250	1.242	1.248
	Metabolism of terpenoids and polyketides	0.005	1.043	1.069	1.089	0.987
	Endocrine system	0.682	0.605	0.630	0.625	0.575
	Transport and catabolism	0.726	0.241	0.193	0.216	0.286
	Endocrine and metabolic disease	0.051	0.232	0.242	0.256	0.204
	Cancer: specific types	0.021	0.055	0.058	0.070	0.046
	Infectious disease: viral	0.005	0.054	0.056	0.069	0.044
	Circulatory system	0.024	0.000	0.000	0.003	0.000
	Signaling molecules and interaction	0.637	0.000	0.000	0.000	0.000
**KEGG-level 3**	Fatty acid degradation	0.001	0.002	0.016	0.012	0.001
	Synthesis and degradation of ketone bodies	0.001	0.034	0.072	0.056	0.025
	Photosynthesis - antenna proteins	0.002	0.001	0.010	0.005	0.000
	Tetracycline biosynthesis	0.002	0.001	0.001	0.006	0.001
	Valine, leucine and isoleucine degradation	0.002	0.020	0.030	0.030	0.011
	Geraniol degradation	0.003	0.000	0.000	0.000	0.000
	Lysine degradation	0.003	0.000	0.000	0.000	0.000

	Chlorocyclohexane and chlorobenzene degradation	0.003	0.000	0.000	0.000	0.000
	Furfural degradation	0.004	0.000	0.000	0.001	0.000
	Tryptophan metabolism	0.005	0.016	0.022	0.023	0.011
	Phenylalanine, tyrosine and tryptophan biosynthesis	0.006	0.000	0.000	0.002	0.000
	Staurosporine biosynthesis	0.007	0.144	0.199	0.167	0.155
	Starch and sucrose metabolism	0.007	0.001	0.000	0.002	0.002
	Lipoarabinomannan (LAM) biosynthesis	0.008	0.125	0.206	0.162	0.118
	Arachidonic acid metabolism	0.008	0.296	0.277	0.264	0.351
	Chloroalkane and chloroalkene degradation	0.008	0.000	0.000	0.001	0.000
	C5-Branched dibasic acid metabolism	0.009	0.015	0.021	0.024	0.008
	Carbon fixation in photosynthetic organisms	0.009	0.015	0.021	0.024	0.008
	Biotin metabolism	0.009	0.000	0.001	0.006	0.000
	Retinol metabolism	0.010	0.000	0.000	0.000	0.000
	Terpenoid backbone biosynthesis	0.011	0.053	0.055	0.059	0.043
	Indole alkaloid biosynthesis	0.011	0.053	0.055	0.059	0.043

	Sesquiterpenoid and triterpenoid biosynthesis	0.012	0.025	0.033	0.026	0.016
	Sulfur metabolism	0.014	0.025	0.034	0.029	0.016
	Isoflavonoid biosynthesis	0.014	0.000	0.000	0.001	0.000
	Flavone and flavonol biosynthesis	0.014	0.112	0.149	0.121	0.106
	Isoquinoline alkaloid biosynthesis	0.015	0.000	0.000	0.000	0.000
	Betalain biosynthesis	0.015	0.000	0.000	0.004	0.000
	Drug metabolism - cytochrome P450	0.015	0.000	0.000	0.004	0.000
	Steroid degradation	0.016	0.000	0.000	0.001	0.000
	Biosynthesis of vancomycin group antibiotics	0.016	0.000	0.000	0.001	0.000
	Biosynthesis of type II polyketide products	0.016	0.000	0.000	0.001	0.000
	Basal transcription factors	0.016	0.000	0.000	0.001	0.000
	Spliceosome	0.016	0.000	0.000	0.001	0.000
	Bacterial secretion system	0.016	0.000	0.000	0.001	0.000
	Homologous recombination	0.016	0.000	0.000	0.001	0.000
	Calcium signaling pathway	0.016	0.000	0.000	0.001	0.000
	p53 signaling pathway	0.016	0.000	0.000	0.001	0.000
	Peroxisome	0.016	0.000	0.000	0.001	0.000

	Apoptosis - multiple species	0.016	0.000	0.000	0.001	0.000
	Cardiac muscle contraction	0.016	0.000	0.000	0.001	0.000
	Dorso-ventral axis formation	0.016	0.000	0.000	0.001	0.000
	Platelet activation	0.016	0.000	0.000	0.001	0.000
	NOD-like receptor signaling pathway	0.018	0.000	0.000	0.000	0.000
	RIG-I-like receptor signaling pathway	0.018	0.000	0.000	0.000	0.000
	Retrograde endocannabinoid signaling	0.019	0.003	0.002	0.008	0.004
	Type II diabetes mellitus	0.021	0.493	0.526	0.516	0.465
	Cushing syndrome	0.022	0.000	0.000	0.000	0.000
	Type I diabetes mellitus	0.022	0.272	0.246	0.246	0.273
	Parkinson disease	0.023	0.055	0.058	0.068	0.046
	Huntington disease	0.024	0.003	0.013	0.009	0.003
	Bacterial invasion of epithelial cells	0.024	0.000	0.000	0.003	0.000
	Pertussis	0.024	0.026	0.016	0.017	0.049
	Yersinia infection	0.025	0.000	0.000	0.000	0.000
	Chagas disease (American trypanosomiasis)	0.025	0.000	0.000	0.000	0.000
	African trypanosomiasis	0.026	0.714	0.850	0.871	0.400

	Hepatitis C	0.026	0.588	0.596	0.607	0.541
	Hepatitis B	0.027	0.000	0.000	0.000	0.000
	Measles	0.030	0.555	0.512	0.527	0.643
	Human cytomegalovirus infection	0.030	0.108	0.113	0.111	0.092
	Influenza A	0.032	0.214	0.273	0.236	0.208
	Kaposi sarcoma-associated herpesvirus infection	0.032	1.288	1.350	1.368	1.027
	Herpes simplex virus 1 infection	0.033	0.950	0.961	1.010	0.881
	Epstein-Barr virus infection	0.034	0.000	0.000	0.001	0.000
	Human immunodeficiency virus 1 infection	0.034	0.000	0.000	0.000	0.000
	Chemical carcinogenesis	0.035	0.000	0.000	0.001	0.000
	Colorectal cancer	0.035	0.000	0.000	0.000	0.000
	Renal cell carcinoma	0.037	0.053	0.055	0.059	0.045
	Bladder cancer	0.039	0.394	0.323	0.342	0.512
	Small cell lung cancer	0.039	0.000	0.000	0.000	0.000
	Hepatocellular carcinoma	0.047	0.088	0.111	0.089	0.101
	Viral myocarditis	0.049	0.153	0.132	0.127	0.156
